# Complexities and Context of Scaling Up: A Qualitative Study of Stakeholder Perspectives of Scaling Physical Activity and Nutrition Interventions in Australia

**DOI:** 10.3389/fpubh.2022.771235

**Published:** 2022-03-28

**Authors:** Harriet Koorts, Jaimie-Lee Maple, Elizabeth Eakin, Mark Lawrence, Jo Salmon

**Affiliations:** ^1^School of Exercise and Nutrition Sciences, Institute for Physical Activity and Nutrition (IPAN), Deakin University, Geelong, VIC, Australia; ^2^School of Public Health, The University of Queensland, Herston, QLD, Australia

**Keywords:** scale up, scaling, physical activity, nutrition, intervention, stakeholder, population health

## Abstract

**Background:**

Scaling up population health interventions is a context-orientated, dynamic and multi-stakeholder process; understanding its influences is essential to enhance future scaling efforts. Using physical activity and nutrition interventions in Australia as case examples, the aim of this paper is to identify core influences involved in scaling up physical activity and nutrition interventions, and how these may differ by context and stakeholder.

**Methods:**

A qualitative study involving semi-structured telephone interviews with individuals representing academic, government and non-government organizations with involvement in scaling up state and national physical activity and nutrition interventions. Interview questions were derived from the WHO report “20 Questions for Developing a Scaling up Case Study”, and mapped against four key principles and five core areas in the WHO ExpandNet framework for scaling up: (1) The innovation; (2) User organization; (3) Environment; (4) Resource team and; (5) Scale up strategy. Data were analyzed thematically.

**Results:**

Nineteen interviews were conducted (government = 3; non-government = 5; and academic = 11 sectors) involving eight scaled up interventions, targeting nutrition (*n* = 2), physical activity (*n* = 1) or a combination (*n* = 5). Most themes aligned to the “Environment”, including: (i) *political* (e.g., personal agendas); (ii) *social* (e.g., lack of urgency); and (iii) *sector/workforce* (e.g., scale up accountability) factors. Themes relating to “Scale up strategy” (e.g., flexibility and evaluation transparency) were next most commonly occurring. Whilst themes were broadly consistent across participants, government participants had a more policy-oriented perspective on the scale up process. Academics discussed a tension between the generation and use of evidence, and the influence of political climates/interest on scale up decisions.

**Conclusion:**

Attributes of the “Environment” and “Scale up strategy” consistently featured as major influences on successful outcomes, while the role of evidence differed greatly between participant groups. A multisector scale up strategy for future interventions may enable the complexities of environmental and political contexts to be incorporated into scale up planning.

## Introduction

Scaling up refers to deliberate efforts to increase the impact of successfully tested interventions, to benefit a greater number of people and to foster policy and programme development on a lasting basis (1). Successful scale up of public health interventions is fundamental to ensuring maximum reach and equitable coverage of interventions to achieve population health improvement. For some important health risk behaviors there has been a continued lack of population-level improvement. Specific to the context of this paper, physical inactivity, unhealthy eating and overweight/obesity, for example, have remained resistant to public health intervention, with levels having reached pandemic proportions worldwide (2, 3). This is observed across the lifespan, with persistently high levels of inactivity, unhealthy eating and overweight/obesity shown from early childhood onwards (4, 5). For example, in Australia, 43% of adults, 29% of children (5–11 years) and 8% of adolescents (12–17 years) achieve government recommended levels of physical activity for improved health (2011–12) (6). In 2019, 67% of Australian adults and one in four Australian children were classified overweight or obese (7). Whilst there are examples of initiatives in Australia that have been scaled up nationally to promote physical activity [e.g., Lift for Life (8)] and a healthy diet [e.g., the Heart Foundation Tick Program (9)]; rates of inactivity (6), and overweight and obesity (10) remain at a level higher than what is currently recommended for health. With regards to physical activity, there has been a significant lack of effective interventions institutionalized within health systems (11), and implementation of large scale obesity prevention approaches have often demonstrated unsustainability (12).

Despite some progress in Australia with the national scale up of interventions targeting improvements in physical activity and a healthy diet; population health impact requires many, sustainably implemented changes targeting all facets of factors influencing these behaviors. For example, physical inactivity and obesity are complex behaviors embedded in many socioeconomic factors. It is not sufficient to simply roll out evidence based interventions targeting one aspect of the problem, all layers of the problem need to be addressed at scale. Scale up, in this instance, is not simply widening the reach of evidence-based interventions. Scaling up is a highly contextually driven process, with scale up decisions and actions embedded in political climates and the goals of agencies invested. A gap in current knowledge is that challenges within the political and social climate of scale-up, and ways of leveraging opportunities when they exist, are rarely reported in the literature (13).

Successful scale up typically requires integrated working with agencies from multiple sectors, beyond just health, and requires commitment from stakeholders both within and outside of government (14). This is important to ensuring the use of appropriate evidence-based programs and potentially increasing the likelihood of sustained investment. It is the “people” involved in the scale-up process that can be central to scale up outcomes. Stakeholders may possess many different “mental models” of how and why scale-up occurs. These mental models can underpin stakeholders' beliefs about why certain outcomes are observed over others, and how these outcomes can be explained. A stakeholder is any person or organization that has an interest in the process of outcomes of the intervention. Previous research on scaled up physical activity and nutrition interventions in Australia has shown that decision-making and perceptions of evidence or intervention legitimacy can, at times, be value-laden and dependent on prior goals and expectations of intervention impact (15). Stakeholders involved in the dissemination or adoption of interventions, such as community-based organizations or governments, can act as the gatekeepers to intervention dissemination or adoption decisions. Understanding the views and beliefs of different stakeholders involved in scale up, including how their perceptions relate to scale up decisions is a critical (16), and yet understudied aspect of scale up (17).

**Figure 1:**
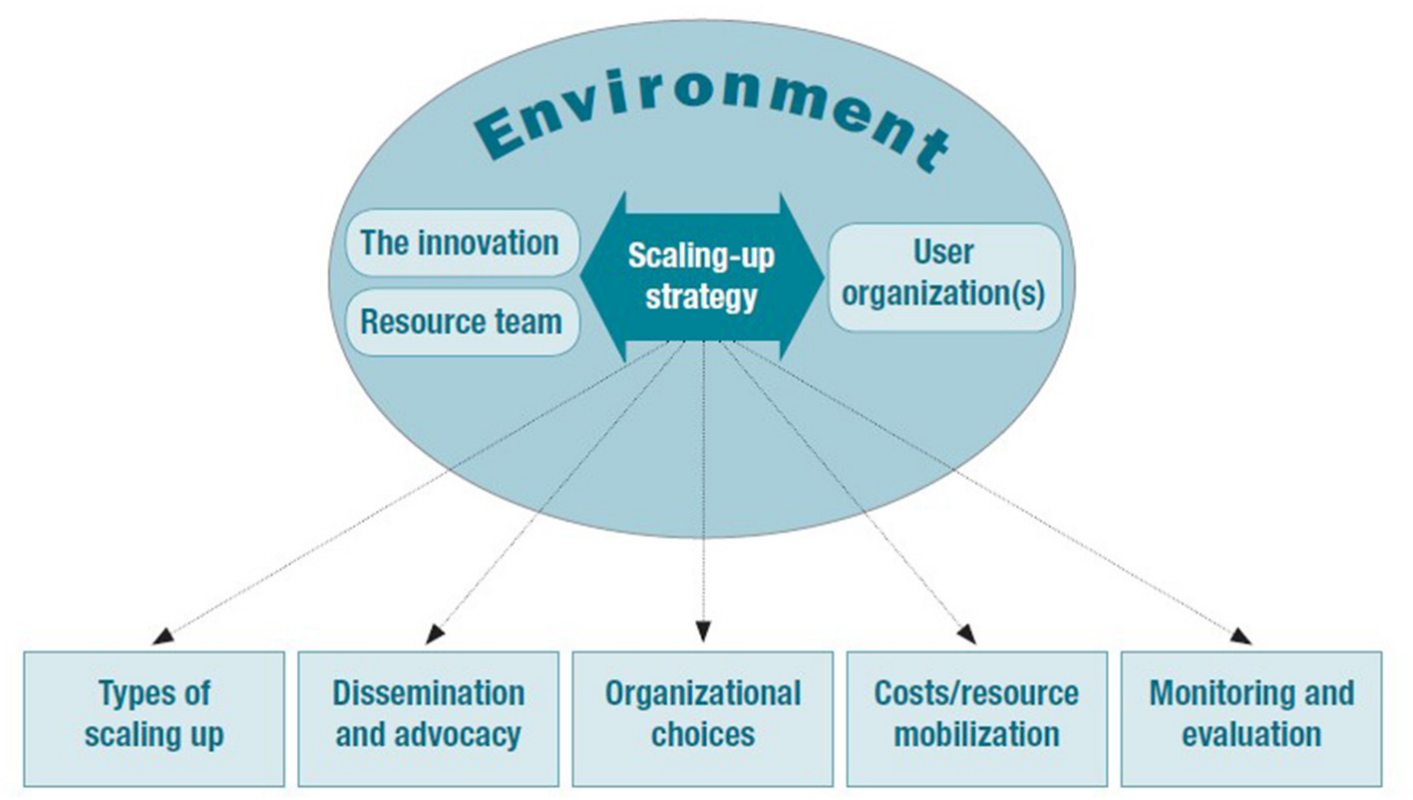
WHO ExpandNet framework for scaling up. Reprinted from: (20).

To accelerate the population health impact of interventions, nonetheless, the World Health Organization (WHO), in collaboration with ExpandNet, has developed multiple resources to support the scale up and translation of evidence-based interventions into practice (18, 19). In particular, the WHO ExpandNet framework for scaling up health service innovations (Figure 1) presents the building blocks for scale up, highlighting the interrelationships among central elements (e.g., attributes of the intervention, implementation strategy and context) and strategic choices involved.

The framework incorporates five core areas, with attributes that are recommended for consideration during scale up: (1) *The innovation (intervention)* (e.g., attributes that increase the likelihood of an intervention being successfully translated such as relevance and compatibility); (2) *User organization* (e.g., organizational attributes such as implementation capacity); (3) *Environment* (e.g., opportunities in the environment to minimize constraints or accelerate institutionalization); (4) *Resource team* (e.g., attributes that increase likelihood of attaining scale up goals, such as effective leadership) and; (5) *Scaling up strategy* (e.g., plans and actions necessary to establish intervention such as advocacy strategies). Whilst the WHO framework for scaling up provides an important road map and compilation of factors involved in scale up globally, its applicability to physical activity and nutrition intervention scale up in the Australian context is unknown.

To address these key gaps in knowledge and explore what the impact is of political and social climates when scaling up, how the perceptions and views of stakeholders influence decision-making processes, and how applicable the WHO framework for scaling up is within the Australian context; in 2018, the Scaling Up InTErvention (“SUITE”) project was conducted. The SUITE project aimed to identify mechanisms underpinning successfully scaled up physical activity and nutrition interventions in Australia and internationally (15), drawing on core areas of the WHO ExpandNet framework (20). This paper presents findings from interviews with key stakeholders involved in scaling up physical activity and nutrition interventions in Australia, from academic, government and non-government sectors. The aim is to investigate factors influencing scale up of state and nationally delivered physical activity and nutrition interventions in Australia, and how these differ based on the perspectives of academics, practitioners and policy makers involved.

## Methods

### Study Design

The study used a qualitative design involving semi-structured telephone interviews with individuals representing academic, government and non-government sectors, involved in scaling up physical activity and nutrition interventions. Scaled up interventions were identified via expert consultation (*n* = 14 subject matter experts), database (e.g., EBSCO) and gray literature searches (*via* Google). A full description of the intervention search strategy has been published previously (15). Included interventions targeted improvements in nutrition (*n* = 2), physical activity (*n* = 1) or a combination of both (*n* = 5); described in Table 1. Collectively, interventions had been implemented in five different implementation settings, targeted populations across the lifespan (although six of the eight targeted children directly or families of children aged 3–13 years), had been delivered in all six Australian states, and included those that were still ongoing in implementation (*n* = 5) or had ceased (*n* = 3) (Table 1). The WHO ExpandNet framework for scaling up (20) was used to inform the interview schedule and the analysis of data. As the aim of this paper was to understand individuals' experiences of and perceived influences on scale up *processes*, as opposed to attributes or features of the unique intervention, we only include data relevant to four of the five framework areas: the Environment, User organization, Resource team and Scale up strategy.

**Table 1:** Descriptive characteristics of scaled up interventions.

**Intervention name**	**Intervention type**	**Target outcome**	**Population and setting**	**Scale up time frame**	**Scale up level**
Go4Fun (21)	After school obesity prevention program	PA & Nutrition	Children aged 7–13 years above a healthy weight, Community settings	2009–ongoing	State (NSW)
Jamie Oliver's Learn Your Fruit and Veg (22)	Community-based program to increase food skills, knowledge and confidence	Nutrition	Children aged 3–12 years, Community settings	2018–ongoing	National
Live Lighter (23)	Educational mass media campaign	PA & Nutrition	Adults, mass media and social media	2012–2015	State (WA, VIC, ACT & NT)
Munch and Move (24)	Training and resources for early childhood educators	PA & Nutrition	Children aged 0–5 years, Early childhood education and care services	2013–ongoing	State (NSW)
OPAL (Obesity Prevention and Lifestyle) (25)	Community development and social marketing	PA & Nutrition	Children through families, Community-based	2009–2017	State (SA)
PEACH (Parenting Eating and Activity for Child Health) (26)	Community-based multi-component group educational sessions	PA & Nutrition	Families with overweight/obese children aged 5–11 years, Community settings	2013–2016	State (QLD)
Physical Activity 4 Everyone (27)	Whole-school physical activity program	PA	Adolescents, Disadvantaged secondary schools	2017–ongoing	State (NSW)
Stephanie Alexander Kitchen Garden (28)	School-based food education program	Nutrition	Primary schools	2005–ongoing	National

*Information in the table relates only to the scale up period for each intervention. Interventions are listed alphabetically and do not correspond to the intervention number provided in illustrative quotes. PA, Physical Activity; Australian states, SA, South Australia; QLD, Queensland; NSW, New South Wales; WA, Western Australia; ACT, Australian Capital Territory; NT, Northern Territory*.

#### Positionality Statement

As in all research, it is helpful to understand our positionality and, therefore, our lens on the data. In conducting this study we operated from the epistemological perspective of social constructivism in that we recognized our inevitable position in the data collection, analysis and interpretation. Although it was not possible to remove ourselves from these procedures we sought to manage our presence in the data in two ways. Firstly, through continual self-reflection on how our worldviews shaped the research. Secondly, by being comprehensive in presenting all data that were collected and then being transparent in reporting how the data were analyzed and reported.

### Participants and Recruitment

Using publically available information on the eight scaled up interventions, key personnel associated with each program were contacted for participation in the study. Participants included key stakeholders grouped according to three levels: (1) academia (University-based academics responsible for designing/testing/evaluating the intervention); (2) government (policy-makers/civil servants involved in government adoption and/or implementation of the intervention) and; (3) non-government (stakeholders in Non-Government Organizations [NGOs], industry or community-based organizations that had a significant role in the scale up process). Participants were eligible for inclusion in the study if they had been either: (i) the principal investigator or a senior chief investigator during intervention development or testing, and/or have knowledge/experience of scaling up process (e.g., participated in decision-making processes related to scale up); (ii) involved in the decision-making process to adopt, fund or roll-out and/or sustain implementation of the intervention at scale, and/or (iii) involved in the process of scaling up the intervention (e.g., funded research/implementation, actively supported or advocated to government for adoption/implementation of the intervention at scale).

Recruitment occurred in two phases. *Phase 1* involved a purposive sampling technique to identify participants named in publications/reports associated with each intervention as identified from online searches. Potential participants were contacted via email and/or telephone and invited to participate in the study. *Phase 2* involved a snowball sampling technique, with Phase 1 participants asked to identify additional individuals who played a significant role in scale up. Participants were asked to forward on the recruitment email or contact details of the research team, to those individuals/organizations directly. To protect participant confidentiality, all “additional” participants identified during Phase 2 recruitment were instructed to confirm their interest to participate by contacting the Principal Investigator. We intended to recruit a representative for each intervention from each of the three stakeholder groups, however, we were unable to do so. This is potentially due to staff turnover given the lengthy time required for scaling up. All participants wishing to take part were emailed information on the study, a Plain Language Statement (PLS), and an individual and organizational consent form.

### Procedure

One-on-one semi-structured telephone interviews were conducted during April to June 2018. Interviews lasted up to 1 h and were audio recorded and transcribed verbatim for later analysis. Individual and organizational signed consent was obtained prior. To ensure interpretation of the interview data was coherent, on completion of the data analysis phase participants were invited *via* email to provide feedback on their quotes for inclusion in the manuscript, and the qualitative theme they related to. Based on feedback, we improved the grammar of two quotes relating to one participant. This grammatical amendment did not alter the meaning of the quote in any way.

### Measures

An interview schedule was developed to explore the drivers underpinning implementation outcomes at scale and how these differed by academics, and those working in government or non-government organizations. Interview questions (*n* = 25) were derived from the WHO report “20 Questions for Developing a Scaling up Case Study”, and the key principles and areas for consideration in the WHO ExpandNet framework for scaling up (20). Table 2 presents application of the four key areas of the WHO ExpandNet framework during interviews.

**Table 2:** Application of the four core areas of the WHO ExpandNet framework for scaling up in interviews.

**WHO core area**	**^a^Description**	**Example interview question**
Environment	Multiple conditions and institutions external to the user organization fundamentally affect the process and prospects for scaling up. The social, cultural, political and economic context in which scaling up takes place has substantial impact on the other elements of the framework.	“*Did the political context at the time the intervention was “rolled-out” affect your efforts or any resources that were needed for scale up?*”
User organization	The institutions or organizations that seek or are expected to adopt and implement the intervention on a large scale. Can include a public sector health service system, a non-government organization or alliance, a network of private, commercial sector providers or a combination of such institutions	“*Were any strategies developed, either by the research team or stakeholders, to ensure the target settings had the capacity to actually implement what was required of them?*”
Resource team	Individuals and organizations that seek to promote and facilitate wider user of the intervention. The resource team serves as a catalyst for change and provides guidance and technical assistance to the deliberate efforts to utilize the innovation on a large scale. Can include researchers, program managers, trainers, service providers, community representatives, reproductive health advocates and policy-makers	“*Was there a role for policy advocates? Were they used to promote the intervention within government or to make decisions on wider roll-out in general?*”
Scale up strategy	Plans and actions necessary to establish the intervention in policies, programs and service delivery. Includes efforts used by the resource team and approaches by the user organization as it responds to these efforts.	“W*hat were the advantages and/or disadvantages to the approach [centralized or decentralized or both] taken in scaling up?”*

a *Core area definitions sourced from Simmons and Shiffman (1)*.

### Analysis

Interview data were thematically analyzed following the methods described by Braun and Clarke (29), and transcripts were coded by participant group (government, non-government and academics). Coding and theme development was firstly deductive, guided by the study aims (20), followed by an inductive approach that was directed by content of the data (30). To prevent any potential bias from researchers with expertise in the topic area, one researcher (JLM) was employed on the project specifically to conduct the data analysis and interpretation of themes for the results, which JLM drafted. JLM is a qualitative researcher who does not specialize in scale-up research, and was not involved as part of the broader research team.

Firstly, interview transcripts were repeatedly read by JLM (Phase I) and then data were coded into subcategories (e.g., consistency of financial support) within NVivo12 software (QSR international) (Phase II). Major categories were created by grouping similar codes/subcategories together (Phase IV). Candidate (i.e., preliminary) themes were identified and reviewed through linking common ideas (categories/subthemes; Phase V). During Phase VI (analysis and write up), themes were linked to direct quotes and presented with participants occupation group. Consistent with Braun and Clarke that prevalence does not necessarily indicate the importance of the theme, inclusion was based on the extent to which the theme helped expand on knowledge to answer the research question, rather than simply occurrence within the data. As one method of researcher triangulation, during interviews, the interviewer (HK) made detailed notes on key points, with suggested codes and key themes summarized at the conclusion of data collection. To further ensure reliability of coding, a second method of researcher triangulation was employed where two researchers (HK, JLM) independently coded a subsample of transcripts (*n* = 5), and then met to discuss and resolve any discrepancies in coding. Researcher triangulation was achieved when there was consensus between JLM and HK on interpretation of the qualitative data.

## Results

**Figure 2:**
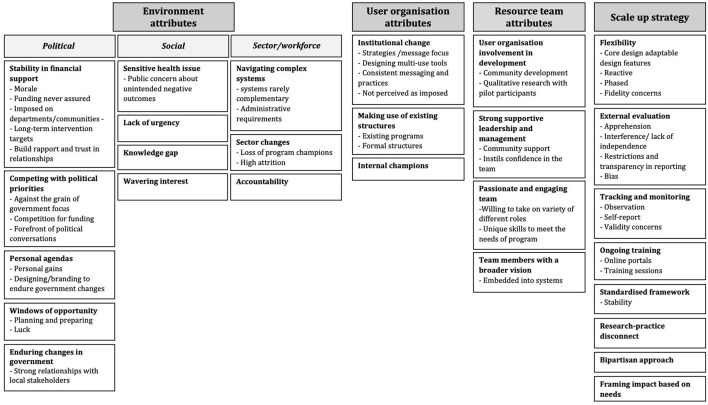
Major themes and subthemes from interviews with individuals involved in scale up. Themes grouped by core areas (dark gray boxes) of the WHO ExpandNet framework for scaling up (13). Bold text indicates a main theme; subthemes listed underneath where applicable.

Nineteen participants (*n* = 3 government; *n* = 5 non-government; *n* = 11 academia) took part in an interview. Participants represented all eight interventions included in the study, however, representation across participant groups varied by intervention. Additional File 1 and Table 1 present descriptive information on participants and the eight scaled up interventions, respectively. Participants' total time involved in the included interventions ranged from <1 year <15 years. Figure 2 depicts how the 27 themes, and 45 subthemes, relate to the core influences on scaling up. To ensure participant confidentiality, interventions listed in Table 1 are presented alphabetically and do not correspond to the intervention number order provided in the illustrative quotes. Although there were some themes unique to participant groups, reflecting their organizational role or specific involvement in scale up, overall, themes were consistent across groups and intervention types.

### Environmental Influences on Scale Up

Political, social and sector/workforce factors were the major environmental influences on scale up. Political factors included competing with political priorities and enduring changes in government, personal agendas and obtaining stability in funding. Social factors included interest and response of the public toward the health issue, which fundamentally impacted the “appeal” for stakeholders to support scaling up. Sector/workforce factors included structural and delivery setting changes that impacted the process and feasibility of scaling up.

#### Political Factors

Stability in financial support for the duration of scale up was mostly attributed to the “perfect political climate” or “luck”. Stability in funding enabled the establishment of large, long-term intervention targets and trusting relationships within the community:

“*… so fickle and fragile* [funding]. *You know I never thought we would lose federal money ‘cause I thought it was so contractually well connected, but evidently not”* (Government 1, Intervention 7)

Academic participants in particular expressed that funding instability was often as a result of investment promises from high profile politicians who then ceased further involvement. The result was interventions feeling “imposed” on departments/communities with inadequate resources and infrastructure to deliver:

“*…the broader infrastructure* [to support scale up] *really wasn't considered because of the initial basis that it* [the intervention] *was set up, which was really this Prime Minister having a pet program that was imposed on the* [name of government department]” (Academic 1, Intervention 1)

The non-government participants discussed that the abundance of causes/interventions being pitched and the finite amount of funding available, was seen to make the success of scale up very difficult if the issue was not already on the forefront of the political conversations. Participants described this as an almost insurmountable challenge, feeling at a loss with ways of overcoming the popularity of other health issues. All groups discussed competition with political priorities, and a change of government was seen as a particular hurdle for interventions with a narrative on “prevention” rather than “cure”:

“*A fact that prevention is seen as such a non-permanent part of the Australian healthcare system is just like, it's more depressing the longer that you've worked in it…you can talk about all these other aspects of scaling up as much as you like, but until healthcare systems embed prevention within their core business, and that it's not seen as a discretionary thing that can come and go depending on the whim of the government or the budget at the time, you're not going to really get very far.”* (Academic 4, Intervention 2)“*we need to be able to shore ourselves up to ride through a government that is more interested in cure than prevention. And that's very hard work…So if we can just ride through one or two seasons of a cure government, we're pretty sure that the prevention government will come back and help us”* (Academic 1, Intervention 1)

Windows of opportunity was a strong theme discussed by non-government participants, and that success depended on the right political climate. Despite planning for this climate, “luck” was a key element to scale up success:

“*Some of them* [outcomes from advocacy strategies] *were fortuitous for sure. Like finally getting into one education minister's sight who just got the vision immediately. That was luck”* (Non-Government 1, Intervention 1)

Academic participants expressed frustration with political drive taking preference over an evidence-based approach:

“*I know many, many, many things that are evidence based with many trials and very strong, but without that political drive, very difficult to get things going. And then you've got other things where the evidence is quite poor, yet the political drive is strong, and it's those things that end up getting implemented. So the strong political drive will outweigh evidence, that's my experience.”* (Academic 1, Intervention 8)“*as scientists or as people who are evidence based, what we want is for decision makers and politicians to make decisions based on evidence. But we know that they don't* [only use evidence]…*And there's lots of other things that are influencing them. And I don't even know that we know what they are half the time. But they're there”*. (Academic 1, Intervention 2)

Navigating personal agendas was a strong theme discussed by all participant groups. Participants expressed that the intervention needed to be consistent with the current government's election commitments and not viewed as continuing the work of the previous government. Participants explained that having the right stakeholders in power was integral to the ultimate success of the intervention, as personal agendas had a strong influence on decision making processes:

“*I hate to say it; self-interest tend to govern most of these conversations* [decisions about scaling up]” (Non-government 1, Intervention 6)

In summary, major themes relating to political factors included: “stability in financial support,” “competing with political priorities,” “personal agendas,” “windows of opportunity” and “enduring changes in government”. Stability in funding was attributed to luck or a perfect political climate, and the challenges of competing with many other health issues. The right political climate/window of opportunity and stakeholder was discussed as influencing decision-making, and in particular having an intervention that was consistent with the current government's election commitments.

#### Social Factors

Influences in the social environment were predominantly discussed by academic and non-government participants. Interventions that were seen as targeting “sensitive” health issues, such as obesity, meant ensuring community engagement and ownership was often difficult. Such health issues could also be perceived by the public as leading to negative outcomes (e.g., creating stigma around children's weight):

“*The main kind of hesitation really for people* [potential stakeholders/advocates] *was about when you referred to obesity and shaming people who were overweight and the risks of that, so we had a lot of talking to do about that.”* (Non-Government 1, Intervention 7)

If the health issue was perceived as less “urgent” compared to others, it was often a challenge to gain community interest and time investment:

“*It's a slow issue* [obesity]*, there's no crisis moment, the community is not necessarily angry about it. I think they're all things were all thinking about a bit more.”* (Non-Government 1, Intervention 4)

Non-government participants also discussed the large knowledge gap and additional hurdles experienced when scaling interventions that reached low socioeconomic groups:

“*What we're seeing, particularly in lower socioeconomic schools, is an alarming knowledge gap…they don't consume fresh fruit and veg…they're not coming from homes that are particularly food literate.”* (Non-Government 1, Intervention 6)

Participants described that varying levels of knowledge amongst those scaling interventions was assumed, whereas planning for innovative ways of translating information was found to be integral. Participants discussed the importance of obtaining a bidirectional social/political interest. As with political environments, community interest was also viewed as “coming in waves” depending on the popularity of the health issue:

“*Concern about childhood obesity comes in waves… so it* [the timing of scale up] *was sort of, if you like, the crest of the obesity wave… So I think sort of the passing of that wave made it less in front of people's minds.”* (Academic 1, Intervention 4)

Major themes relating to social factors included: “sensitive health issues,” “lack of urgency,” “knowledge gap,” “wavering interest.” Gaining community buy-in for an intervention targeting a sensitive health issue was described as challenging, and in addition, if it was an issue perceived as less urgent by the community. There were knowledge gaps regarding how to target low socioeconomic groups, and that community interest came in waves. This wavering interest was described as relating to the popularity of the health issue among the public.

#### Sector/Workforce Factors

All participants referred to the fact that scaling up often involved navigating systems. The resulting administrative burden this led to caused resources and time to be wasted, which was a significant hindrance to the process. The complex systems involved in scaling up were rarely complementary to one another, both between and within sectors:

“*Because universities are* [a] *huge bureaucracy, governments are a huge bureaucracy and they've both got their rules that are hard to kind of bend”* (Academic 4, Intervention 2)

The impact of sector changes was another consistent theme, particularly in regards to the loss of program champions, which ultimately led to decreased attention and loss of support for the intervention. Researchers discussed scaling up in environments that were particularly susceptible to high attrition, and this subsequently resulted in wasted resources re-training new employees:

“* a couple people left the organisation who were strong advocates and the originators of the intervention. As they left the level of support began to wane…so many resources started being withdrawn and it was difficult to keep it going in the end.”* (Government 2, Intervention 7)

Government participants discussed ‘accountability' and experiencing an unwillingness to claim responsibility for addressing certain health issues. These decisions were often influenced by risk aversion or the actions of other jurisdictions:

“*I think some of them* [local government areas] *wondered whether it was their business to be looking at what they considered to be a health issue.”* (Government 2, Intervention 7)“*…there were a lot of discussions between the state* [departments] *saying, well if they're out* [federal government] *we're out.”* (Government 1, Intervention 7)

Sector/workforce themes included: “navigating complex systems,” “sector changes” and “accountability.” The complex systems involved in scaling up were described as adding administrative burden and inefficiencies to the scale up process. Sector changes, such as staff turnover, also impacted consistent buy-in for the intervention. Accountability and responsibility for the intervention varied at a local level, which could relate to perceptions of the health issue being targeted and state/federal support.

### User Organization Influences on Scale Up

Participants acknowledged that successful scale up may require institutional change and discussed the importance of using existing structures and internal champions. Participants described an increased likelihood of successful and sustainable scale up, if institutional change was achieved. Specifically, if the focus of the intervention was less on fidelity of implementation and more on embedding new strategies and messages within the user organization. If interventions were perceived as imposed on user organizations, participants discussed that the likelihood of institutional change was reduced:

“*in many cases they* [intervention requirements] *were being imposed on* [the setting and staff] *who already had their agenda…And so I can imagine in some instances that would have generated hostility if not at minimum wanting to wish it away. Let me get on with my job.”* (Academic 1, Intervention 1)

Non-government participants in particular discussed that intervention applicability to a broad range of teaching objectives/other areas beyond the intervention target; increased the likelihood of it becoming embedded in practice. Whereas, academic participants discussed the importance of achieving culture change through consistent messaging and practices across the user organization. Identifying existing, broader community programs and formal structures within user organizations promoted intervention acceptance and sustainability of scale up. Some participants discussed that embedding their intervention within already established initiatives, and tailoring it to fit within existing structures, led to rapid community engagement, that otherwise may not have been achieved:

“*it was a really clever design, to place it squarely as part of* [a bigger initiative]. *That just makes the community engagement so easy. I've had other roles where you spend about three quarters of your time chasing people that you should be working with, to see if you can get any interest.”* (Non-Government 1, Intervention 4)

In some instances, internal champions were a conscious change from the original trial (external change agent) to the scaled up intervention (internal change agents) that was seen as key for both acceptability and sustainability of scale up:

“*the funding was sent to the* [setting] *for them to identify an existing…member…who could drive the intervention. And I think that was of benefit…the* [setting] *champion already knew, knew who they had to go to approach to get something happening.”* (Academic 2, Intervention 5)

Key themes for user organization influences included: “institutional change,” “making use of existing structures” and “internal champions.” Institutional change that promoted embedding the intervention within existing systems was described as key to sustainable scale up. Identifying existing formal structures within user organizations that aligned with the intervention, and using champions embedded within the organizations, were also described as promoting scale up sustainability in the delivery setting.

### Resource Team Influences on Scale Up

Participants discussed involving user organizations in the development of interventions, and characteristics of the leadership and support team members to sustain advocacy and intervention implementation:

“*it wasn't a program that was made up outside of an education environment then distributed. It was made and developed with the educators who we wanted to influence. So we were able to learn from them about what was the best approach.”* (Non-Government 1, Intervention 6)

Non-government and government participants also discussed the importance of community development to ensure engagement and ownership for sustainability of scale up. In addition, strong, supportive leadership was described as not only increasing the likelihood of community support, but also instilling confidence within the program team.

“*…arguably, the higher order reason for success, was the leadership, that we had a state manager* [name]*, who was phenomenally gracious with* [their] *power and* [their] *control. Where* [they] *100% backed the team in. And again because we were so encouraged to take risks we would make mistakes, because we were so ambitious about what we were trying to achieve, and how we were going to try and achieve it.”* (Government 1, Intervention 7)“*having a really good person managing the program and the roll out is really critical. And that person has to have, you know, very good skills in developing partnerships, in writing, in persuading, in making a team come together, working extraordinarily hard. You know, it's a huge ask. And there are some difficult times. So I think that's really critical.”* (Non-Government 1, Intervention 7)

Participants discussed the importance of employing a team based on their unique skills, passion and engagement needed for the specific intervention being scaled up:

“*The importance of charisma in your team…the work of the team was to be that always uber positive presence in any context, and that we would be very engaging, very charismatic, very real and very grounded, more than happy to sit in the dirt with grandma, more than happy to frock up and put on a suit and go play a political role.”* (Government 1, Intervention 7)

Academic participants discussed the importance of involving individuals who had the ability to think broadly about program implementation and how to achieve system-level embeddedness to ensure sustainability:

“*we've got the right people around the table advising on, you know, what the future system looks like, of how it needed to be embedded, as well as, well you know what implementation science evidence we need to support that it's made a change”* (Academic 1, Intervention 5)

This was discussed in regards to both individual team members and effective collaboration between policymakers and academics.

In summary, key themes related to “user organization involvement in development,” “strong supportive leadership and management,” “passionate and engaging team,” and “team members with a broader vision.” Involving targets users in intervention development was described as essential, and was linked to ownership and engagement within the community. Specific characteristics of the scale up team (e.g., being engaging) were described as important for system-level embeddedness and thus sustainability of the intervention.

### Influences of the Scale Up Strategy

Participants referred to the advantages of being reactive to situations and windows of opportunity, as opposed to solely prescriptive scale up planning:

“*Being reactive rather than proactive is sometimes a bonus, sometimes an advantage…we have spent lots of time over the years in trying to be more proactive and trying to put frameworks together and trying to plan things. Only to have them fall over at the last minute because whatever we had planned for just didn't exist by the time we got there… we don't know when the opportunities are going to come. And we've got to be sort of elastic enough to be able to absorb them when they do”* (Non-Government 1, Intervention 1)“*I think in the absence of having a situation in which you can invest long term in a programme and incrementally improve it in a structured way,…there's no way it can keep going because you'll always have to be contextually adapting it.”* (Academic 4, Intervention 2)

All participants highlighted that this implementation feature was essential to adapt to different environments, however, some participants additionally discussed the importance of adhering to a logic model (a “standardized framework”) with prescribed features that acted as a cornerstone to all implementation decisions. These established features were seen as creating stability regardless of the unpredictability of the scale up environment and ensuring consistency across program sites:

“*The framework that we developed, the logic model… it gave us a very strong touchstone where even though we might be taking risks, or being risky, in a couple of instances, it gave us that – it's like you can run back to mum, if that makes sense.”* (Government 1, Intervention 7)

Among academic participants, many of whom had experience in the evaluations of the scaled up interventions, they described evaluations often being met with apprehension among funders due to the potential for “exposing flaws” or less desirable findings:

“*I would say there has been some resistance to us coming in, because it may identify some flaws, because we would have a scientific approach… But it has limited the scientific nature of what we have been able to do, because we're limited. We're restricted. So it's sort of like, OK bring us into the tent, but be very careful about what we're allowed to do and say.”* (Academic 1, Intervention 8)

Participants described restrictions on external evaluations, with “good practice” often being compromised by the scope of the evaluation, and the lack of validated measures/limited resources available. Transparency in the evaluations was often controversial due to interference by program funders:

“*there was also a bit of unnecessary interference in the sense that…the evaluation was meant to be completely independent in terms of the whole project. And that was important scientifically to really find out well what works, what doesn't work, how it's all going and what are the elements that are making it work, et cetera. And there was continual interference in terms of the funders in wanting to input how things were done and wanting to sort of change things on the run and so on. And that at times was problematic because it just created a bit of tension between the evaluation team. Not that it stopped anything happening but…at times was a little bit annoying, put it that way.”* (Academic 4, Intervention 7);

Some academic participants described a bias in the publication of evaluation results, swayed toward predominantly positive outcomes, whereas program limitations were often hidden if reported at all:

“*the biggest issue identified was the lack of reach of the program. That was very hidden and very buried in the very back of the paper. And when I have tried to explore this issue…to try and see how we could improve it…it was certainly put to me, that being very overt with that hidden problem of reach, was not acceptable in a publication, basically.”* (Academic 1, Intervention 8)“*my view is this data is being gathered from the public purse, it should be made widely available. And that was also the view of the funders, they thought that but they somehow… their thought process didn't sort of… it wasn't equateable to actually making it happen…I mean the whole* [name of intervention] *evaluation data is meant to be up on a website. It's meant to be free for anyone to come and dig into, but it's not.”* (Academic 4, Intervention 7)

Academic participants referred to the disconnect between the pursuit for scientific rigor and “hard outcomes,” and the realities of what is feasible when adaptations are often required when implementing interventions in practice. This conflict was often overlooked when planning evaluations of scaled interventions, and led to less positive perceptions of study results:

“*if a government is wanting an evidence-based programme and they want to… they want to us to prove that* [it works]*, then they need to respect that* [the intervention changes] *to a certain extent as well. I mean, you just can't throw the baby out with the bath water*. (Academic 4, Intervention 2)“*The scientific advisory committee, for instance, was very high level academic and quite pompous at times as to what they expected. And I think they didn't necessarily understand the challenges and difficulties and everyday nuts and bolts of carrying out both the intervention and evaluating it. So they were really interested in hard core outcomes, you know changing BMI z-scores and that sort of thing, and making sure that we got a sufficient sample to be able to say something, and all of the rest of it. And so there was a bit of a disconnect if you like between that and when you're really in the field and on the ground, things are different and you have to adapt a lot.”* (Academic 4, Intervention 7)

The majority of government and non-government participants discussed evaluation in terms of tracking and monitoring activities, and adoption of program principles. This was mostly achieved through observation and self-report measures:

*There wasn't any formal external evaluation on that scale up. But what there was monitoring of the school activity… we would get the reports from the schools… including the school visits that we did, which were really, really great for capturing the program in action visually…We could really get people to see what was going on. We used and still use content from those reports to point to different benefits, impacts, improvements, successes, challenges, constantly.”* (Non-Government 1, Intervention 1)“*We had a* [name of resource] *that people would write what they covered* [in their sessions] *and that was in an effort to capture the program content… as a measure of fidelity… But you know, it's kind of biased. It's self-reported* [by the implementer]. *And you don't really know if they did that”* (Academic 1, Intervention 2)

Academic participants expressed concerns with the validity of relying on these methods. In some instances participants expressed that the need for a formal evaluation became apparent partway through implementation in order to demonstrate the effectiveness of scale up. In these instances, evaluation was set up retrospectively:

“[if we had the chance again] *we would have probably put evaluation in at the very beginning. Like if we had had enough money and time and thought we would have had someone monitoring and evaluating the whole time because we are now backtracking a lot.”* (Non-Government 1, Intervention 6)*It was a classic community-generated program where you go and do something and then you think about the evaluation later rather than setting it up so that there was good baseline data to work with. So it* [the evaluation] *was reflective in that sense.”* (Academic 1, Intervention 1)

However, building strategies for ongoing training and learning was considered as essential for successful scale up. Online portals and regular training sessions were seen as valuable design features that enabled ongoing education for user organizations:

“*having that* [training] *portal ready to go and explain the importance of it* [the intervention] *and walk them* [the implementers] *through it, so I think that is key”* (Academic 2, Intervention 5)

Profiling and framing impact of the intervention based on needs, so it appealed to a wide range of audiences was described as critical to government buy-in and ongoing support. In particular, gaining the support of influencial advocates who could champion the initiative, both publically and privately, was considered critical to scale up success:

“*Critical elements for success definitely are public profile and getting the influencers to see the program in action. The two evaluations have also been incredibly key to be able to demonstrate to bureaucrats that there is a formal evaluation process around it. So again, it's just speaking to everybody's different needs.”* (Non-Government 1, Intervention 1)

Participants referred to the importance of influencing multiple political parties (a “bipartisan approach”) as a strategy to maintain the relevance, sustainability and potential ongoing buy-in into the intervention:

“*We probably should have always tried to influence the party that was in power and the party that was not in power.”* (Non-Government 1, Intervention 1)

Although acknowleded retorspectively, none of the participants referred to succesfully achieving this.

In summary, key themes relating to influences of the scale up strategy included: “flexibility,” “external evaluation” “tracking and monitoring”, “ongoing training”, “standardized framework”, “research-practice disconnect”, “framing impact based on needs”, and a “bipartisan approach.” Whilst the importance of reactivity to windows of opportunity and use of a scale-up framework was recognized, their value depended on the context/intervention being scaled. The transparency of external evaluations could be compromised due to involvement from program funders and a percieved risk of reporting of less desirable findings. Essential elements of scale-up included strategies for ongoing training, and framing the intervention impact to appeal to a wide range of stakeholders and political parties (e.g., a bipartisan approach).

### Contrasting Experiences by Those Working in Government, Non-government and in Academia

In general, themes representing all four of the framework domains were consistent across participant groups and individuals had commonalities in their experiences. However, overall, political and social environmental influences were predominantly discussed among government participants. As expected, government participants had a more policy-oriented perspective on the scale up process. However, government participants' perceptions of policy influence typically centered on ways that political advocacy could be used to align scaling up with windows of political opportunity. Whilst participants representing non-government and academic organizations discussed political influences, these were framed in terms of sustainability of funding and political support. Themes relating to user organization and resource team attributes were also broadly consistent across all participant groups. Although, academic participants discussed in more depth the need for a team who had a broader vision for the program and the ability to understand the importance of embedding the program within systems. In terms of scale up strategies, participant experiences were fairly similar in regards to ways of enhancing implementation at scale, however, government participants notably valued “standardized” frameworks to guide scale up. Flexibility was discussed as an essential component by most participants, however, academic participants emphasized difficulties when navigating fidelity issues when adopting a flexible approach.

Unlike other major themes relating to the WHO framework, factors relating to monitoring and evaluation were notably different between participant groups. Whilst the role of evidence was discussed by all participant groups, for government and non-government participants it tended to be framed in terms of monitoring of activities and as an advocacy tool for further funding or implementation. Academic participants, however, referred to the tension between the multiple roles evidence has to play when scaling initiatives. This was in terms of both environmental factors (such as evidence competing with political priorities), and as part of the scale up strategy (evidence generated needed to be “fit for purpose” or framed in a way to amplify only certain outcomes). Some academic participants described concerns over the transparency of the evaluation process and questioned the ethical nature of the boundaries within which they were permitted to collect and publish data. In particular, there were concerns raised regarding the limited capacity to publish outcomes. This was described in terms of a conflict between their desire to report outcomes important to inform population health and future implementation (e.g., weaknesses in the scale up processes or less successful elements) vs. outcomes that were part of a contractual agreement. A common outcome to funding restrictions was the ceasing of evaluations or modifications to core data collected, and as such, there was a perception that this could lead to misleading program evaluations and publication bias.

## Discussion

The purpose of this study was to investigate factors influencing scale up of state and nationally delivered physical activity and nutrition interventions in Australia, and how these differ based on the perspectives of academics, practitioners and policy makers involved. Our findings contribute to addressing three key gaps in current knowledge regarding the impact of political and social climates when scaling interventions, how the perceptions of stakeholders influence decision-making, and the extent that the WHO ExpandNet framework (20) for scaling up is applicable to the Australian context. In terms of applicability of the WHO framework, qualitative themes represented all four areas of the framework, although, consistently across all participants; dominant influences on the adoption and sustainability of scaled up interventions related to framework attributes of the *Environment* (such as the political, social and workforce factors), and concepts related to the *Scale up strategy*.

In terms of the factors influencing scale up, and role of political and social climates, we found that political environmental factors were consistently described as having the greatest influence on the initiation of scale up and its subsequent sustainability. Consistent with previous research, this included a lack of stability in funding, difficulties competing with existing and changing political priorities, and navigating personal agendas of politicians. High-level political support is one of the most important factors for intervention scalability (11), and adequate and stable funding for global nutrition interventions is an established driver for scale up (31). Policy “enabling” environments and governance (e.g., stability in funding and investments in capacity) also have an overarching role in the utilization of nutrition research (32), and act as a mechanism for adoption and at-scale implementation of physical activity and nutrition interventions by government (15). The time and costs involved to mobilize the many different stakeholders and sectors involved in scale up (14) cannot be underestimated; political priorities and investments will change. In addition, reasons for the lack of successful scale up of physical activity and nutrition interventions are complex and dynamic, incorporating multiple political, environmental and social factors (15). For example, advocacy and commitment from implementers (33), availability of resources and political will (9), and the values, goals, agendas and world views of those involved (15); can both enhance and impede the uptake and sustainability of interventions at scale. Our findings showed that the influence of political environmental factors on scale up processes went beyond a context-to-outcome relationship often depicted in the literature [i.e., a positive political climate (context) leading to increased buy-in from political advocates (outcomes)]. Rather, in some cases, the political environment was perceived to override all other features associated with successful scaling. For example, a supportive community or strong evidence base could become less relevant if political support was not strong and consistent.

In terms of how perspectives differed across participants, and the influence of stakeholder perceptions on decision making processes, whilst all participants acknowledged the vital role political climates and political interests play during the advocacy of some interventions over others, only academics referred to this in context of a “research-practice disconnect”. Academics discussed a perceived tension between their desire for rigorous evidence generation and political commitment to evidence-informed decision making, with the realities of political agendas that governed scale up decisions. Governments in particular have one of the most important roles to play in coordinating national strategies, at a local, state and federal level, and yet their use of research evidence to inform decision-making processes is varied. For example, a survey of policy-makers identified 95% used local data to inform decision-making and 85% indicated this was most valued. Academics were rarely consulted and research evidence rarely used (34). Whilst all participants discussed that evidence had a role in informing scale up processes; government and non-government participants tended to discuss this in terms of implementation monitoring and as a strategic advocacy tool for ongoing funding for implementation sustainability.

This raises important questions regarding academics' investment in evidence *production* with that of communication and advocacy strategies for evidence *use*, and the relative importance of different areas of scale up frameworks and how they are interrelated. The WHO framework for scaling up emphasizes the interaction between scale up elements and that scale up does not occur in a vacuum, unaffected by external factors. However, it is unclear whether “hierarchies of influence” exist, and whether they should be disaggregated in the scale up literature. For example, in terms of the framework's applicability in this study, one of the core areas we investigated, attributes of the “User organization” were not described as having a major role in influencing scale up outcomes. We do not interpret this as meaning that there is a redundancy in the role of those framework domains, nonetheless. Previous research has suggested a taxonomy of scaling up processes, which differentiates “political scaling up” from other aspects of the more generalized scale up approach (35). The rationale is that political activities influence the extent of a politically “enabling” scale up environment. Whilst the taxonomy classifies *quantitative* (e.g., expansion in program size), *functional* (e.g., increases in activities) and *organizational* (e.g., increasing organizational capacity) scaling processes; the processes are interrelated. User organization and resource team (i.e., implementer) attributes have been widely studied as a major factor influencing implementation of intervention in practice [e.g., (36)]. Rather, our findings suggest that the role of the user organization may in fact be more pertinent in terms of real-world delivery of interventions in practice, as opposed to during the scale up decision making process more generally. This is potentially due to the fact that the user organization has a more embedded role in adoption and implementation in practice settings.

Other major factors influencing scale up outcomes related to the “Scale up strategy,” which was interrelated to political environmental factors. For example, evaluation data that aligned with stakeholder requirements (“scale up strategy”) was discussed in relation to favorable political advocacy (“environmental attributes”). However, unlike themes relating to “environmental attributes,” which predominantly centered on influences attributable to government and non-government organizations in the community, themes relevant to the scale up strategy included the role of academia and academics as influencing the scaling process. Themes related to flexibility and reactivity to real-world implementation, transparency in evaluation processes, logic models for implementation, and strategic political plans for buy-in. Having a strategic plan for scale up is considered one of the most important influences in public health scaling, and specifically, the availability of monitoring and evaluation data (9). Whilst our findings support this in principal, academic participants described a perceived interference and lack of transparency in intervention evaluations during scale up, with evidence generation and use heavily value laden.

Whilst it is recommended that interventions require planning for scalability (19) and effective leaders to ensure broad implementation (37), many, but not all, of the interventions included in our study included formal plans for scaling up, such as a logic model or framework, and embedded monitoring and evaluation plans. Our findings showed that whilst participants expressed support for planning; reactivity to contextual opportunities and change was perceived a major advantage. Notwithstanding the known benefits of planning tools for implementation and scale up, such as to mitigate potential hurdles when scaling and for strategic targeting of advocates and program champions (20); our findings highlighted additional roles of such tools. They provided a fall-back procedure and structure of “reassurance” in light of “fears of making a mistake” when scaling. It has been suggested that we need a broader conceptualization of scaling up that accounts for uncertainty in the process, and a reframing of how we use and interpret frameworks for scaling up which is currently absent from traditional models and approaches (38). This need not mean that implementation and scale up planning is devoid of flexibility and reactivity, on the contrary, contextual adaptation is a known core facilitator for implementation and scale-up in real-world contexts (14). Rather, our findings highlight that scale-up frameworks and models offer more than simply a blueprint of recommended steps. How successful scale-up is *framed* and depicted in frameworks and models (i.e., descriptions of key steps in the process and anticipated outcomes from these steps) can inform the *role* these resources play during the scale-up process (i.e., how stakeholders use frameworks and models to guide their decision-making).

### Strengths and Limitations

A major strength of this study was the inclusion of a diverse range of interventions, contexts and implementation approaches, to understand scale up processes. Moving beyond a case study approach, whereby one scaled up intervention is retrospectively evaluated, by integrating multiple, multilevel perspectives on program adoption, implementation and sustainability outcomes; the potential generalisability of our results is enhanced. Many participants, particularly those working in government and non-government organizations, also had experience of scaling interventions in areas other than physical activity and nutrition, and could draw on these experiences during interviews. This has important implications in terms of strengthening the generalisability of our results to other interventions, contexts and areas of public health scale up. Qualitative data analysis also enabled us to gain a much deeper insight into scale up processes, moving beyond published evidence on scale up outcomes to elucidate relationships and nuances between factors that otherwise might not have been possible.

There are, however, a number of limitations. Whilst we had more than one stakeholder group representing each intervention included, we were unable to recruit participants representing all three stakeholder groups for every intervention. As such, it is unknown how findings may differ with a broader representation of participants for each of the scaling up contexts. In addition, participants' length of time involved in the intervention varied across participant groups and programs. Although several participants had been working for their organizations for 5 years or less and this may raise questions about their seniority and/or authority, the study eligibility criteria required participants to have experience and knowledge of the scale-up process as a lead investigator or funder, and/or have been involved in scale-up decision-making processes. For the purpose of this study, we explored influences on the scale up *process* as opposed to influences of the specific intervention characteristics (that would have been captured under the WHO framework core area “Intervention attributes,” which was excluded from this study). As such, the role and impact of environmental factors and strategy for scaling may simply be more explicit than those related to specifics of the interventions, given the study focus. Irrespective of this nonetheless, the themes identified from interviews were broadly consistent both within and across participant groups, including across intervention type; increasing confidence in the results. Our inclusion criteria required that programs were described in an adequate level of detail to determine eligibility. Reliance on the quality of publically available information on each intervention means that other potentially “eligible” interventions may have been missed. To mitigate the potential for this, we adopted a three phase search strategy, which included peer-reviewed and gray literature searches, and physical activity and nutrition expert consultation (15). Likewise, the search strings used to identify scaled up interventions were developed and tested in consultation with a research librarian, which we adapted from those used by Reis et al. (11). This strategy was adopted to encourage consistency with the broader scale up literature, however, again, there is the potential that some relevant interventions may have been missed due to variations in the terminology used.

### Recommendations for Future Scale Up Research and Practice

To influence outcomes of future scale up efforts in practice:

- Approaches may need to be reorientated from primarily emphasizing intervention characteristics when planning for scale up (e.g., intervention replicability), to other factors related to political, social and environmental domains.
- A reframing of how we approach and understand scale up may also be needed, to help shift current scale up paradigms. A reframing from perceptions of linearity when scaling interventions in public health, to one of complexity that accounts for interrelated factors (38), and relative importance of different constructs (15).
- A multisector scale up strategy may lessen the impact of challenges navigating complex systems, between and within sectors, when scaling in public health. A scale up infrastructure that includes multiple levels and sectors of accountability may also improve the impact and efficiency of scaling processes (14, 33, 39).

To advance the field of research more broadly:

- Scale up frameworks may need to account for “hierarchies of influence” to reflect the specific vs. holistic influence of some aspects of scaling over others.
- Scale up frameworks may also need to provide a distinction between priorities of influences relating to different domains of scaling up (such as the political environment) over others (such as intervention characteristics to enable broad reach) where possible. This may assist those wishing to scale interventions to incorporate multiple logic models and frameworks to accommodate the many forms and functions that scaling up can take.
- Scale up tools and resources may also need to address the relationship between factors perceived to be highly influential but less modifiable in practice. For example, despite the known importance of political environmental factors, political support has been perceived as a feature far less feasible to influence when scaling in physical activity (11).

## Conclusion

By drawing on concepts within the WHO ExpandNet framework for scaling up, our findings address important gaps in the public health scale up literature; identifying how elements known to impact scale up are relevant when scaling in practice. The in-depth qualitative data enabled us to tease apart how influences were interrelated and where hierarchies of influence exist. Irrespective of the type of intervention, level of government investment, or scale up approach; attributes of the environment and the strategy for scaling consistently featured as major influences on successful scale up. Although evidence was identified as an important element during scale up, the role of evidence and transparency of how it was used, differed greatly between those working in academia, government and non-government organizations. Nonetheless, as scaling up involves many complexities and interrelated elements, all domains of the WHO ExpandNet framework remain relevant when scaling in public health. Our findings underpin important recommendations for future scale up approaches and areas for research, and contribute toward disentangling the complexity of scale up processes in physical activity and nutrition, and in public health more generally.

## Data Availability Statement

The datasets used and/or analysed during the current study are available from the corresponding author on reasonable request.

## Ethics Statement

Ethical approval was obtained from Deakin University Human Research Ethics Committee (12_2018). All participants provided individual and organizational informed signed consent to participate. Participants were also provided the opportunity to review any of their quotes selected for inclusion in published outputs. All methods were carried out in accordance with relevant guidelines and regulations. The patients/participants provided their written informed consent to participate in this study.

## Author Contributions

HK conceptualized and designed the study and leading writing of the manuscript. J-LM conducted the qualitative data analysis and drafted the results. JS, EE, and ML contributed to the SUITE study design. JS and ML to the intervention screening process. All authors revised the manuscript for intellectual content, read, and approved the final draft.

## Funding

This study was funded by a Deakin University Faculty of Health project grant in 2018. JS was supported by a National Health and Medical Research Council Leadership Level 2 Fellowship (APP 1176885).

## Conflict of Interest

The authors declare that the research was conducted in the absence of any commercial or financial relationships that could be construed as a potential conflict of interest.

## Publisher's Note

All claims expressed in this article are solely those of the authors and do not necessarily represent those of their affiliated organizations, or those of the publisher, the editors and the reviewers. Any product that may be evaluated in this article, or claim that may be made by its manufacturer, is not guaranteed or endorsed by the publisher.

## Abbreviations

WHO: World Health Organization
SUITE: Scaling Up InTErventions
NGO: Non-Government Organizations
PLS: Plain Language Statement
PA: Physical Activity.

## References

[1] Simmons R Shiffman J. Scaling up health service innovations: a framework for action. In: Simmons RFP Ghiron L editor. Scaling Up Health Service Delivery. Geneva: World Health Organization (2007).

[2] Kohl HW Craig CL Lambert EV Inoue S Alkandari JR Leetongin G. The pandemic of physical inactivity: global action for public health. Lancet. (2012) 380:294–305. 10.1016/S0140-6736(12)60898-8 22818941

[3] Swinburn BA Sacks G Hall KD McPherson K Finegood DT Moodie ML. The global obesity pandemic: shaped by global drivers and local environments. Lancet. (2011) 378:804–14. 10.1016/S0140-6736(11)60813-1 21872749

[4] Venn AJ Thomson RJ Schmidt MD Cleland VJ Curry BA Gennat HC. Overweight and obesity from childhood to adulthood: a follow-up of participants in the 1985 Australian schools health and fitness survey. Med J Aust. (2007) 186:458–60. 10.5694/j.1326-5377.2007.tb00997.x 17484707

[5] Telama R. Tracking of physical activity from childhood to adulthood: a review. Obes Facts. (2009) 2:187–95. 10.1159/000222244 20054224 PMC6516203

[6] Australian Bureau of Statistics. Australian Health Survey: Physical Activity, 2011–12. Canberra, ACT: Australian Bureau of Statistics (2013).

[7] Australian Institute of Health and Welfare. Australian Institute of Health and Welfare 2019. Overweight and Obesity: An Interactive Insight. Canberra, ACT: AIHW (2019).

[8] Nettlefold L Naylor PJ Macdonald HM McKay HA. Scaling up action schools! BC: how does voltage drop at scale affect student level outcomes? A cluster randomized controlled trial. Int J Environ Res Public Health. (2021) 18:5128. 10.3390/ijerph18105182 34068235 PMC8153156

[9] Bulthuis SE Kok MC Raven J Dieleman MA. Factors influencing the scale-up of public health interventions in low- and middle-income countries: a qualitative systematic literature review. Health Policy Plan. (2019) 35:219–34. 10.1093/heapol/czz140 31722382 PMC7050685

[10] Australian Institute of Health and Welfare. Poor diet. Canberra, ACT: AIHW (2019).

[11] Reis RS Salvo D Ogilvie D Lambert EV Goenka S Brownson RC. Scaling up physical activity interventions worldwide: stepping up to larger and smarter approaches to get people moving. Lancet. (2016). 10.1016/S0140-6736(16)30728-0 PMC5193005 27475273

[12] World Health Organization. Consideration of the evidence on childhood obesity for the Commission on Ending Childhood Obesity: report of the ad hoc working group on science and evidence for ending childhood obesity. Geneva: World Health Organization (2016).

[13] ExpandNet Management Systems International World Health Organization. 20 Questions for Developing a Scaling-Up Case Study. World Health Organization (2007). Available online at: http://www.expandnet.net/tools.htm

[14] Koorts H Eakin E Estabrooks P Timperio A Salmon J Bauman A. Implementation and scale up of population physical activity interventions for clinical and community settings: the PRACTIS guide. Int J Behav Nutr Phys Act. (2018) 15:51. 10.1186/s12966-018-0678-0 29884236 PMC5994105

[15] Koorts H Cassar S Salmon J Lawrence M Salomon P Dorling H. Mechanisms of scaling up: combining a realist perspective and systems analysis to understand successfully scaled interventions. Int J Behav Nutr Phys Act. (2021) 18:42. 10.1186/s12966-021-01103-0 33752681 PMC7986035

[16] Milat AJ King L Newson R Wolfenden L Rissel C Bauman A. Increasing the scale and adoption of population health interventions: experiences and perspectives of policy makers, practitioners, and researchers. Health Res Policy Syst. (2014) 12:18. 10.1186/1478-4505-12-18 24735455 PMC3996855

[17] Mugo C Njuguna I Nduati M Omondi V Otieno V Nyapara F. From research to international scale-up: stakeholder engagement essential in successful design, evaluation and implementation of paediatric HIV testing intervention. Health Policy Plan. (2020) 35:1180–7. 10.1093/heapol/czaa089 32944754 PMC7810404

[18] World Health Organization. Nine Steps for Developing a Scaling-Up Strategy. Geneva: World Health Organization (2010).

[19] World Health Organization. Beginning With the End in Mind: Planning Pilot Projects and Other Programmatic Research for Successful Scaling Up. Genva: World Health Organization (2011).

[20] World Health Organization. Practical Guidance for Scaling Up Health Service Innovations. Geneva: World Health Organization (2009).

[21] Welsby D Nguyen B O'Hara BJ Innes-Hughes C Bauman A Hardy LL. Process evaluation of an up-scaled community based child obesity treatment program: NSW Go4Fun(R). BMC Public Health. (2014) 14:140. 10.1186/1471-2458-14-140 24512080 PMC3923092

[22] Cooke J Ariss S Smith C Read J. On-going collaborative priority-setting for research activity: a method of capacity building to reduce the research-practice translational gap. Health Res Policy Syst. (2015) 13:25. 10.1186/s12961-015-0014-y 25948236 PMC4455707

[23] Morley B Niven P Dixon H Swanson M Szybiak M Shilton T. Population-based evaluation of the 'LiveLighter' healthy weight and lifestyle mass media campaign. Health Educ Res. (2016) 31:121–35. 10.1093/her/cyw009 26956039 PMC4802349

[24] Lockeridge A Innes-Hughes C O'Hara BJ McGill B Rissel C. Munch & Move: Evidence and Evaluation Summary. North Sydney NSW: NSW Ministry of Health (2015).

[25] Richards Z Kostadinov I Jones M Richard L Cargo M. Assessing implementation fidelity and adaptation in a community-based childhood obesity prevention intervention. Health Educ Res. (2014) 29:918–32. 10.1093/her/cyu053 25214513

[26] Croyden DL Vidgen HA Esdaile E Hernandez E Magarey A Moores CJ. A narrative account of implementation lessons learnt from the dissemination of an up-scaled state-wide child obesity management program in Australia: PEACH™ (Parenting, Eating and Activity for Child Health) Queensland. BMC Public Health. (2018) 18:347. 10.1186/s12889-018-5237-8 29534700 PMC5851159

[27] Sutherland R Campbell E Lubans DR Morgan PJ Okely AD Nathan N. ‘Physical Activity 4 Everyone' school-based intervention to prevent decline in adolescent physical activity levels: 12 month (mid-intervention) report on a cluster randomised trial. Br J Sports Med. (2016) 50:488–95. 10.1136/bjsports-2014-094523 26359346 PMC4853531

[28] Block K Gibbs L Staiger PK Gold L Johnson B Macfarlane S. Growing community: the impact of the stephanie alexander kitchen garden program on the social and learning environment in primary schools. Health Educ Behav. (2012) 39:419–32. 10.1177/1090198111422937 22167317

[29] Braun V Clarke V. Using thematic analysis in psychology. Qual Res Psychol. (2006) 3:77–101. 10.1191/1478088706qp063oa

[30] Joffe H Yardley L. Content and Thematic Analysis. In: Marks D Yardley L editors. Research Methods for Clinical and Health Psychology. London: Sage Publications (2004).

[31] Gillespie S Menon P Kennedy AL. Scaling up impact on nutrition: what will it take? Adv Nutr. (2015) 6:440–51. 10.3945/an.115.008276 26178028 PMC4496740

[32] Menon P Covic NM Harrigan PB Horton SE Kazi NM Lamstein S. Strengthening implementation and utilization of nutrition interventions through research: a framework and research agenda. Ann N Y Acad Sci. (2014) 1332:39–59. 10.1111/nyas.12447 24934307

[33] Spicer N Bhattacharya D Dimka R Fanta F Mangham-Jefferies L Schellenberg J. ‘Scaling-up is a craft not a science': catalysing scale-up of health innovations in Ethiopia, India and Nigeria. Soc Sci Med. (2014) 121:30–8. 10.1016/j.socscimed.2014.09.046 25306407

[34] Oliver KA de Vocht F. Defining ‘evidence' in public health: a survey of policymakers' uses and preferences. Eur J Public Health. (2017) 27(suppl. 2):112–7. 10.1093/eurpub/ckv082 26163467

[35] Gillespie S. Scaling Up Community-Driven Development: A Synthesis of Experience. Washington, DC (2004).

[36] Damschroder LJ Aron DC Keith RE Kirsh SR Alexander JA Lowery JC. Fostering implementation of health services research findings into practice: a consolidated framework for advancing implementation science. Implementat Sci. (2009) 4:1–15. 10.1186/1748-5908-4-50 19664226 PMC2736161

[37] McCannon C Berwick DM Massoud M. The science of large-scale change in global health. JAMA. (2007) 298:1937–9. 10.1001/jama.298.16.1937 17954547

[38] Koorts H Rutter H. A systems approach to scale-up for population health improvement. Health Res Policy Syst. (2021) 19:27. 10.1186/s12961-021-00679-0 33648525 PMC7919988

[39] Øvretveit J Garofalo L Mittman B. Scaling up improvements more quickly and effectively. Int J Qual Health Care. (2017) 29:1014–9. 10.1093/intqhc/mzx147 29177491

