# Supplementary material for: Complexities and Context of Scaling Up: A Qualitative Study of Stakeholder Perspectives of Scaling Physical Activity and Nutrition Interventions in Australia
Source: Front Public Health. 2022 Mar 28;10:771235. doi: 10.3389/fpubh.2022.771235 (PMC8995799; doi:10.3389/fpubh.2022.771235)
Supplement: Supplementary file 1 [file Data_Sheet_1.docx]

Additional File 1: Descriptive information on participants

|  | **Government (n=3)** | **Non-government (n=5)** | **Academics (n=11)** |
| --- | --- | --- | --- |
| **Age (range)** | 35-39 years (1)  55-59 years (1)  45-49 years (1) | 30-34 years (1)  40-44 years (1)  45-49 years (1)  50-54 years (1)  60+ years (1) | 30-34 years (1)  40-44 years (2)  45-49 years (4)  60+ years (4) |
| **Sex** | Female (1)  Male (2) | Female (4)  Male (0)  Prefer not to say (1) | Female (10)  Male (1) |
| **Time in current organisation** | <1 year (1)  1-5 years (2) | <1 year (1)  1-5 years (3)  6-10 years (1) | <1 year (1)  1-5 years (4)  6-10 years (2)  11-15 years (1)  16-20 years (1)  21-25 years (1)  >25 years (1) |
| **Total time involved with intervention** | >1 <6 years (1)  >6 <10 years (2) | <1 year (1)  >1 <6 years (2)  >6 <10 years (1)  Prefer not to say (1) | ^a^<1 <6 years (9)  >6 <10 years (1)  >10 <15 years (1) |

Number of years in Row ‘Time in current organisation’ need not correspond to Row ‘Total time involved with intervention’, as participants could have worked in multiple organisations during their time involved with the intervention.
